# Central Neuromuscular Reactivation Using Digital Mindfulness and Motor Imagery Intervention for Chronic Knee Pain in Patients With Obesity: Prospective, Single-Center Study

**DOI:** 10.2196/82270

**Published:** 2026-01-26

**Authors:** Jerome Murgier, Bertrand Garet, Sonja Murgier, Guillaume Zunzarren

**Affiliations:** 1 Aguilera Private Clinic, Rue de l'estagnas, Biarritz, 64100, France, +33 0973032124; 2 St Vincent's Hospital Melbourne, melbourne, Victoria, Australia

**Keywords:** obesity, knee pain, motor imagery, central neuromuscular reactivation, digital mindfulness

## Abstract

**Background:**

Chronic musculoskeletal pain, particularly in patients with obesity, poses significant challenges due to increased pain sensitivity, reduced mobility, and systemic inflammation. Obesity aggravates mechanical constraints on the joints and increases systemic inflammation, exacerbating certain medical conditions and making conventional therapeutic approaches less likely to succeed. Conventional therapies often show limited efficacy, necessitating innovative approaches.

**Objective:**

This pilot study evaluated the short-term effectiveness of a mindfulness and motor imagery-based intervention delivered via an app (SAS YUZIT), on the pain, functionality, and quality of life of patients with obesity and chronic knee pain.

**Methods:**

A prospective, single-center study was conducted over 1 month by including 30 patients (BMI >30 kg/m^2^) experiencing chronic knee pain (≥3 months) who did not need surgery. Patients underwent two video-guided motor imagery sessions, focusing on neuromuscular reactivation. Functional scores, including the Single Assessment Numeric Evaluation (SANE) score, Knee Injury and Osteoarthritis Outcome Score (KOOS), and 36-Item Short Form Health Survey (SF-36) score, were assessed before and after intervention. Paired *t* tests were used to analyze score improvements, with *P*<.05 deemed significant.

**Results:**

Significant improvements were observed across all parameters. The mean overall SANE score improved by 51% (*P*<.001). The minimal clinically important difference was therefore approximately 6.7 points. The observed mean improvement (25 points) exceeded this threshold by more than threefold, indicating a clinically meaningful improvement in functional status. The mean (SD) KOOS score increased by 56% (*P*<.001) from 40.89 (16.7) to 63.79 (14.6). The mean (SD) SF-36 scores showed substantial enhancements in both physical (from 39.7 [20.8] to 65.9 [18.8]; +66%; *P*<.001) and mental components (from 47.2 [23.1] to 64 [21]; +36%; *P*<.001). Patient satisfaction with this method was rated 4.5/5, and no adverse effects were reported.

**Conclusions:**

Video-guided motor imagery demonstrated significant efficacy in reducing pain and improving the functionality and quality of life of patients with obesity and chronic knee pain. By targeting central neuromuscular circuits through guided visualization exercises, this non-invasive intervention addresses central activation dysfunction, a proposed novel concept in neuromuscular disorders that originates from hypotheses derived from clinical observations. This interesting concept requires further exploration to ensure its neurophysiological validation through future studies with larger sample sizes, control groups, and long-term follow-ups, all of which could explore CAD as a therapeutic target.

## Introduction

Chronic musculoskeletal pain poses a major limiting factor to functional capacity and can negatively impact quality of life, particularly in patients with obesity (BMI >30 kg/m^2^) [1,2]. A higher BMI is associated with increased pain sensitivity, reduced mobility, and multiple comorbidities that complicate management paradigms. Obesity aggravates mechanical constraints on the joints and increases systemic inflammation, exacerbating certain medical conditions and making conventional therapeutic approaches less likely to succeed [3-5]. The impact on the knee joint is particularly significant, and it is well demonstrated that being overweight is a risk factor for chronic knee pain and even degenerative progression [6-8]. Chronic musculoskeletal pain carries a significant physical burden on patients but also represents a significant overall cost to society [3].

Traditional methods, such as drug treatments, weight loss, and regular physical practices, show limitations in this population [9,10] due to the increased risk of complications, subtherapeutic responses, and significant financial costs [8,9]. Chronic pain impacts all areas of the patient’s life and often leads to global societal, personal, and professional complications [11,12]. Resolving this problem is therefore a public health issue.

Therapeutic approaches do not always take into account the central impact that pain has on the patient’s overall motor control [11,13], particularly in patients with obesity who have more difficulties with postural or neuromotor performance [14]. Modern therapies target central rehabilitation aimed at using brain neuroplasticity to improve patient conditions [15-17]. Emerging digital rehabilitation tools are now available to patients, such as augmented reality, various biofeedback devices, mental imagery with low frequency sounds, or smartphone apps [18].

This pilot study describes and evaluates a new method for treating knee pain in patients with obesity to complement existing therapies. This innovative, non-invasive therapy combines mindfulness and motor imagery [19]. This specific form of guided motor imagery known as neuromuscular reactivation (NMR) can be undertaken via an app. It consists of NMR sessions, designed to activate central neuromuscular circuits without requiring physical movement [20].

The gradual development of NMR occurred in response to neuromuscular activation deficits after an ACL tear [21]. Scientific research has proven that these deficits are of central origin and that it is potentially interesting to target this area to improve patient outcomes [22]. Despite these advances, there remains limited evidence regarding the clinical efficacy of app-based neuromotor interventions in cohorts of patients with obesity.

This pilot study aimed to evaluate the short-term effectiveness (1 month) of NMR on perceived pain, functionality, and quality of life of patients with obesity and chronic knee pain. The primary objective was to evaluate the method’s impact on pain (Single Assessment Numeric Evaluation; SANE) and quality of life (36-Item Short Form Survey; SF-36) scores at a minimum 1-month follow-up. Secondary objectives were to explore improvements in objective functional scores (patient-reported outcome measures) and to assess patient satisfaction after the intervention. We hypothesized that a standardized motor imagery-based intervention would significantly improve the different clinical parameters in a patient group at high risk of chronicity.

## Methods

### Study Design

This was a prospective, observational, monocentric, continuous study conducted over a 1-month period at a medical center with consecutive patient inclusions. The inclusion criteria were as follows: patients had to have a BMI greater than 30 kg/m^2^, be of legal age, and present with chronic knee pain (>3 months) with a magnetic resonance imaging scan showing no need for surgery. Patients were recruited in clinics from January 2025 to March 2025.

Data were collected by the practitioner during the consultations through the use of single-blinded questionnaires. The standardized forms used were intended to reduce collection bias. During the initial assessment, complete demographic data were collected, as well as the following functional scores: the SANE score (global and for the painful limb), the SF-36 score, the KOOS score, and patient satisfaction.

The intervention consisted of two video-guided motor imagery sessions, delivered via an app (Yuzit), 3 weeks apart. Patients were guided to visualize specific movements by associating them with optimal muscle sensations, without performing any physical movement per se.

### Session Description

The sessions were conducted in a quiet room, where each patient was seated alone with a digital tablet. They would then launch the first video, lasting 53 minutes, designed to guide a structured session of motor imagery.

This video followed a precise sequence: It starts with an explanation of the session’s progression (1 min), followed by a mindfulness phase (3 min 20 s). Then, for each exercise, a visual demonstration of the movement is given, followed by guided visualization, with eyes closed, focusing on internal muscle sensations, without any physical execution.

The first session included 12 exercises, performed with bilateral visualization, following the same educational structure. The movements demonstrated in the video were as follows: (1) hip extension, leg straight, in a supine position; (2) hip flexion, in a prone position; (3) locking against resistance (pressure applied to the plantar aspect of the foot) in triple flexion, lying on the back; (4) dorsal and plantar flexion of the foot, lying on the back; (5) fist squeezing (clenched fist under the knee), lying on the back; (6) abduction/adduction of a straight leg, lying on the back; (7) knee extension, in a seated position; (8) unipedal stance (balance on one leg); (9) rising onto tiptoes with locking in a unipedal stance; (10) unipedal squat; (11 )“schuss”, that is, shifting body weight to one leg in semi-flexion; (12) sitting down on a chair.

The second shorter session (approximately 30 min), focused on the painful side (or bilateral in cases of bilateral pain), following the same structure (visual understanding followed by visualization). It included 6 unilateral exercises: (1) leg raise with the leg straight, lying on the back; (2) triple flexion, lying on the back; (3) knee flexion, lying on the stomach; (4) standing position on both feet, then on one foot (unipedal stance); (5) going up and down stairs; (6) sitting position.

The same functional scores were collected 1 month after the last session during a follow-up visit, in addition to the quality scores. All patients completed the exercises with practitioner oversight intermittently throughout the duration of the two video sessions.

The Standards for Reporting Qualitative Research was followed to write this paper.

### Ethical Considerations

This study was conducted in accordance with the ethical principles of the Declaration of Helsinki. Ethical approval was obtained from the French ethics committee of Saint-Étienne (approval number 2025-A01476-43). Where applicable, informed consent was obtained from all participants prior to their inclusion in the study. Data were anonymized and de-identified to ensure confidentiality of all participants. No compensation was provided to any participant.

### Statistical Analysis

Continuous variables were described using means, SDs, and ranges. The normality of changes before and after the intervention was assessed using the Shapiro–Wilk test. Data were analyzed using paired one-tailed *t* tests to compare pre- and post-intervention scores. A significance threshold of *P*<.05 was used. The minimal clinically important difference was estimated using a distribution-based method, calculated as 0.5 × SD of the change scores. The SD of change was computed from baseline and follow-up SDs, assuming a pre–post correlation coefficient (*r*) of 0.7, in accordance with common practice for functional outcome measures.

## Results

### Patient Characteristics

A total of 30 patients were included in the study. Their clinical and demographic characteristics are described in Table 1.

**Table 1.** Clinical and demographic characteristics (N=30).

Characteristic	Value
Age, years	
Mean (SD)	48.4 (12.7)
Range	22‐75
Weight, kg	
Mean (SD)	92.9 (16.8)
Range	68‐110
Height, cm	
Mean (SD)	168 (11.3)
Range	150‐190
BMI, kgm^2^	
Mean (SD)	33.1 (3)
Range	30.1‐42.2
Sex, n (%)	
Male	12 (40)
Female	18 (60)

### Changes in Functional and Qualitative Scores

The mean (SD) overall SANE score was 49 (18.4) before the intervention versus 74 (14.3) after 1 month. The mean improvement was 51% (*P*<.001). The mean (SD) SANE score for painful limbs before surgery was 41.9 (19.6) versus 71.16 (15.2) after 1 month. The mean improvement was +69% (*P*<.001). Based on a distribution-based approach, the SD of change scores was estimated using an assumed pre–post correlation of 0.7, yielding an SD of 13.3. The minimal clinically important difference was therefore approximately 6.7 points.The observed mean improvement (25 points) exceeded this threshold by more than threefold, indicating a clinically meaningful improvement in functional status.

The mean (SD) KOOS score before surgery was 40.89 (16.7) versus 63.79 (14.6) after 1 month, with a mean improvement of +56% (*P*<.001). The SF-36 quality of life score showed a significant mean (SD) improvement of +66% in the physical component (*P*<.001), increasing from 39.7 (20.8) to 65.9 (18.8). The mental component of the score also significantly improved, increasing from 47.2 (23.1) to 64 (21), representing an improvement of +36% (*P*<.001), as shown in Table 2.

The mean satisfaction score assigned by patients to the method was 4.5/5. It should be noted that no side effects were found in this study.

**Table 2.** Changes in functional and qualitative scores.

	Score before intervention (out of 100)	Score after intervention (out of 100)	Improvement (%)
SANE^a^ (global)	49	74	+51
SANE (affected knee)	41.9	71.1	+69
KOOS^b^/HAGOS^c^	40.9	63.8	+56
SF-36 PCS^d^	39.7	65.9	+66
SF-36 MCS	47.2	64	+36

a SANE: Single Assessment Numeric Evaluation.

b KOOS: Knee Injury and Osteoarthritis Outcome Score.

c HAGOS: Copenhagen Hip and Groin Outcome Score.

d SF-36 PCS: 36-Item Short Form Health Survey Physical Health Component.

e SF-36 MCS: 36-Item Short Form Health Survey Mental Health Component.

## Discussion

### Principal Findings and Comparison With Previous Works

The hypothesis of this study was confirmed. The results show significant improvements in pain, functionality, and quality of life scores after an intervention based on mindfulness and motor imagery via an app. This method, by targeting central neuromuscular circuits, represents an innovative, non-invasive approach suited to complex population, such as patients with obesity and chronic knee pain.

To our knowledge, there is no literature addressing the problem of chronic knee pain in patients with obesity using alternative treatments targeting the central origin of these symptoms and relying on cerebral neuroplasticity [23]. This central pathophysiology has been partly explained by the analysis of anterior cruciate ligament trauma.

Knee trauma causes a neuromodulatory reflex loop that reduces quadriceps activation capacities and increases that of the hamstrings [24]. The resulting corticomotor deficits have been explored by several authors [25-27] who investigated corticomotor excitability, corticospinal reflex excitability, motor unit sizes, and voluntary action in a series of patients undergoing ACL surgery. Zunzarren et al [21] found out that this inactivation induced by knee trauma also affected the contralateral quadriceps, thus leading to the hypothesis that changes in the neuromuscular response involve phenomena that go beyond a simple corticomotor reflex [28] and finds its origin in the cortex. Piskin et al [29] confirmed this phenomenon and demonstrated that it can affect both legs. These neuromotor consequences have significant repercussions as patients experience a double trauma (the injury and the reflex). A growing number of studies [22,30] highlight the need for rehabilitation targeting central stimulation, using patients’ cerebral neuroplasticity [31]. The objective is to restore the patient’s overall capacity to recruit all the muscle fibers via a central action (through global neuromuscular activation), before stimulating short corticomotor circuits, and thus, obtain maximum efficiency [32]. This complex phenomenon can affect any joints [22]. These findings are essential because they pave the way for very significant changes in the management of knee injuries and pain by integrating the notion of central neuromuscular damage [30]. This has led doctors and physiotherapists to formulate novel rehabilitation techniques, notably to mitigate persistent muscular deficits.

To achieve optimal efficacy, it appears that a “central disinhibition” is necessary. Other NMR therapies (stimulating the corticospinal stage) have recently emerged, notably a technique using mental representation and proprioception associated with low-frequency sounds, with promising results [16,29]

In this study, the significant increase in the SANE score (from 41.9 to 71.2) and the KOOS score (from 40.9 to 63.8) illustrates a significant reduction in perceived pain and functional improvement. These results are particularly encouraging given the limitations associated with obesity, including greater pain intensity and a poorer response to conventional interventions.

Improvements in the physical component of the SF-36 (+23.5 points) reflect a better perception of physical capacity, while the increase in the mental component (+19.4 points) reflects a reduction in the psychological impact. These results highlight the importance of considering neuro-cerebral dimensions in the management of musculoskeletal pain.

### The Potential Role of Central Activation Dysfunction

#### Overview

One of the major lessons learned from this study is the validation that muscle activation disorders are not exclusively related to peripheral mechanisms but also to central disorders. This is the reason why the term “arthogrenic muscle inhibition” [31], which is widely accepted as an explanation for muscle deficits in post-traumatic or inflammatory conditions, is insufficient to describe the whole phenomenon.

We introduce the concept of central activation dysfunction (CAD) to describe a central disruption of neuromuscular circuits. Unlike arthogrenic muscle inhibition, which relies on joint inhibitory reflexes, CAD involves an alteration of central sensorimotor mechanisms. This phenomenon could be explained by a disruption in the cortical representation of the injured muscle, a functional disconnection between the motor centers of the central nervous system and peripheral motor neurons and a chronic negative conditioning, where persistent pain permanently alters muscle perception.

These muscle activation disorders, which we group together under the term CAD, are based on empirical observations from clinical practice. Although this concept has not yet been validated by in-depth neurophysiological studies, it offers a coherent hypothesis to explain muscle activation deficits that don’t come with an obvious articular or peripheral origin. We identified several possible mechanisms contributing to these disorders, grouped into three categories: physical trauma, consequences of surgical interventions, and chronic pain and psychosocial factors.

#### Physical Trauma

Physical trauma like muscle injuries, whether intrinsic (eg, tears) or extrinsic (eg, direct trauma such as heavy crutches), or even some joint trauma, can lead to persistent muscle activation deficits. In these cases, pain likely acts as a central factor disrupting the connections between the brain and the affected muscles.

#### Consequences of Surgical Interventions

Postoperative outcomes, although they address structural pathologies, often leave unexplained functional sequelae. These deficits, traditionally considered purely mechanical or inflammatory, could also be due to a central activation defect. This appears to be particularly common when pain persists in the absence of identifiable mechanical complications.

#### Chronic Pain and Psychosocial Factors

In young patients with chronic pain, we have observed that muscle activation deficits are rarely associated with identifiable structural abnormalities. These disorders could be linked to several factors, including physical trauma (acute or long-standing), with the associated pain potentially disrupting central control mechanisms; rapid growth requiring a centralized reappropriation of the body, which is often disrupted; and emotional issues such as depression, family conflicts (eg, parental divorce), or psychological trauma. These contexts directly influence the central nervous system, contributing to functional deficits.

Our hypothesis is based on the idea that, in these situations, pain is not only a symptom but also a major disruptor of central sensorimotor circuits. While this hypothesis remains to be validated by further research, it appears rather logical. By proposing the concept of CAD, we offer a new perspective to explain why some conventional approaches fail to restore optimal muscle activation. This conceptual framework also paves the way for specific interventions, such as motor imagery.

### Study Limitations

Although the results are promising, this preliminary study has several limitations. First, the low number of patients included in this paper (30 patients) can be seen as decreasing the statistical power of the study; however, the differences found in this study reached statistical significance. Given this is a pilot study, we deemed this to be sufficient to allow preliminary findings to be made. Then, the short follow-up failed to provide long-term data. The long-lasting effect of the method has to be evaluated. Finally, a control group was missing to improve the level of proof of this paper.

### Conclusions

The results of this preliminary study suggest a mindfulness-based method that could be effective in reducing chronic pain and improving quality of life of patients with obesity. By introducing the concept of CAD, this study opens new perspectives for understanding and treating muscle activation disorders. Given the preliminary nature of this study, the broader implications of this research could identify other patient groups who could benefit from this type of treatment. These results warrant further research to refine protocols and better integrate this approach into clinical practice. If adopted, this could prove to be a cost-effective and non-invasive method for rehabilitation. A randomized or sham-controlled trial would be needed to draw conclusions about efficacy and safety.

## Abbreviations

CAD: central activation dysfunction
KOOS: Knee Injury and Osteoarthritis Outcome Score
NMR: neuromuscular reactivation
SANE: Single Assessment Numeric Evaluation
SF-36: 36-Item Short Form Health Survey

## References

[1] Lin I, Wiles L, Waller R (2020). What does best practice care for musculoskeletal pain look like? Eleven consistent recommendations from high-quality clinical practice guidelines: systematic review. Br J Sports Med.

[2] Costa ABP, Machado LAC, Telles RW, Barreto SM (2024). Obesity and the risk of multiple or severe frequent knee pain episodes: a 4-year follow-up of the ELSA-Brasil MSK cohort. Int J Obes.

[3] Bindawas SM, Vennu V (2015). Longitudinal effects of physical inactivity and obesity on gait speed in older adults with frequent knee pain: data from the Osteoarthritis Initiative. Int J Environ Res Public Health.

[4] Walsh TP, Arnold JB, Evans AM, Yaxley A, Damarell RA, Shanahan EM (2018). The association between body fat and musculoskeletal pain: a systematic review and meta-analysis. BMC Musculoskelet Disord.

[5] Jinks C, Jordan K, Croft P (2006). Disabling knee pain--another consequence of obesity: results from a prospective cohort study. BMC Public Health.

[6] Tanamas SK, Wluka AE, Davies-Tuck M (2013). Association of weight gain with incident knee pain, stiffness, and functional difficulties: a longitudinal study. Arthritis Care Res (Hoboken).

[7] Silverwood V, Blagojevic-Bucknall M, Jinks C, Jordan JL, Protheroe J, Jordan KP (2015). Current evidence on risk factors for knee osteoarthritis in older adults: a systematic review and meta-analysis. Osteoarthr Cartil.

[8] Paley CA, Johnson MI (2016). Physical activity to reduce systemic inflammation associated with chronic pain and obesity: a narrative review. Clin J Pain.

[9] D’Arcy Y (2011). Managing pain in obese patients. Nurse Pract.

[10] Felisbino-Mendes MS, Cousin E, Malta DC (2020). The burden of non-communicable diseases attributable to high BMI in Brazil, 1990–2017: findings from the Global Burden of Disease Study. Popul Health Metrics.

[11] Hinwood NS, Casey MB, Doody C (2024). The experiences of people living with obesity and chronic pain: a Qualitative Evidence Synthesis (QES) protocol. PLoS ONE.

[12] Ayis S, Dieppe P (2009). The natural history of disability and its determinants in adults with lower limb musculoskeletal pain. J Rheumatol.

[13] Meng H, O’Connor DP, Lee BC, Layne CS, Gorniak SL (2016). Effects of adiposity on postural control and cognition. Gait Posture.

[14] Wankhede NL, Koppula S, Ballal S (2025). Virtual reality modulating dynamics of neuroplasticity: innovations in neuro-motor rehabilitation. Neuroscience.

[15] Dos Anjos T, Guillot A, Daligault S, Chamoun DM, De Sousa T, Di Rienzo F (2024). Low-frequency sounds combined with motor imagery elicits a transient disruption of force performance: a path to neuromotor reprogramming?. Neuroimage.

[16] Eaves DL, Riach M, Holmes PS, Wright DJ (2016). Motor Imagery during action observation: a brief review of evidence, theory and future research opportunities. Front Neurosci.

[17] Howard CK, Yamada M, Dovel M (2024). An objective assessment of neuromotor control using a smartphone app after repeated subconcussive blast exposure. Sensors (Basel).

[18] Vogt S, Di Rienzo F, Collet C, Collins A, Guillot A (2013). Multiple roles of motor imagery during action observation. Front Hum Neurosci.

[19] Hardwick RM, Caspers S, Eickhoff SB, Swinnen SP (2018). Neural correlates of action: comparing meta-analyses of imagery, observation, and execution. Neuroscience & Biobehavioral Reviews.

[20] Dos Anjos T, Gabriel F, Vieira TD, Hopper GP, Sonnery-Cottet B (2024). Neuromotor treatment of arthrogenic muscle inhibition after knee injury or surgery. Sports Health: A Multidisciplinary Approach.

[21] Zunzarren G, Garet B, Vinciguerra B, Murgier J (2023). Persistence of neuromuscular activation deficit in the lower limb at 3-years of follow-up after ACL reconstruction surgery. Knee.

[22] Dos Anjos T, Guillot A, Kerautret Y, Daligault S, Di Rienzo F (2022). Corticomotor plasticity underlying priming effects of motor imagery on force performance. Brain Sci.

[23] Chmielewski TL, Hurd WJ, Rudolph KS, Axe MJ, Snyder-Mackler L (2005). Perturbation training improves knee kinematics and reduces muscle co-contraction after complete unilateral anterior cruciate ligament rupture. Phys Ther.

[24] Hollman JH, Buenger NG, DeSautel SG, Chen VC, Koehler LR, Schilaty ND (2022). Altered neuromuscular control in the vastus medialis following anterior cruciate ligament injury: a recurrence quantification analysis of electromyogram recruitment. Clin Biomech (Bristol).

[25] Lepley AS, Gribble PA, Thomas AC, Tevald MA, Sohn DH, Pietrosimone BG (2015). Quadriceps neural alterations in anterior cruciate ligament reconstructed patients: a 6-month longitudinal investigation. Scand J Med Sci Sports.

[26] Pietrosimone BG, Lepley AS, Ericksen HM, Clements A, Sohn DH, Gribble PA (2015). Neural excitability alterations after anterior cruciate ligament reconstruction. J Athl Train.

[27] Luc-Harkey BA, Harkey MS, Pamukoff DN (2017). Greater intracortical inhibition associates with lower quadriceps voluntary activation in individuals with ACL reconstruction. Exp Brain Res.

[28] McPherson AL, Schilaty ND, Anderson S, Nagai T, Bates NA (2023). Arthrogenic muscle inhibition after anterior cruciate ligament injury: injured and uninjured limb recovery over time. Front Sports Act Living.

[29] Piskin D, Benjaminse A, Dimitrakis P, Gokeler A (2022). Neurocognitive and neurophysiological functions related to ACL injury: a framework for neurocognitive approaches in rehabilitation and return-to-sports tests. Sports Health.

[30] Dos Anjos T, Gabriel F, Vieira TD, Hopper GP, Sonnery-Cottet B (2024). Neuromotor treatment of arthrogenic muscle inhibition after knee injury or surgery. Sports Health.

[31] Machan T, Krupps K (2021). The neuroplastic adaptation trident model: a suggested novel framework for ACL rehabilitation. Int J Sports Phys Ther.

[32] Sonnery-Cottet B, Hopper GP, Gousopoulos L (2024). Incidence of and risk factors for arthrogenic muscle inhibition in acute anterior cruciate ligament injuries: a cross-sectional study and analysis of associated factors from the SANTI study group. Am J Sports Med.

